# Species and genotypes belonging to *Echinococcus granulosussensu lato* complex causing human cystic echinococcosis in Europe (2000–2021): a systematic review

**DOI:** 10.1186/s13071-022-05197-8

**Published:** 2022-03-28

**Authors:** Adriano Casulli, Alessandro Massolo, Urmas Saarma, Gérald Umhang, Federica Santolamazza, Azzurra Santoro

**Affiliations:** 1 WHO Collaborating Centre for the Epidemiology, Detection and Control of Cystic and Alveolar Echinococcosis, Department of Infectious Diseases, Istituto Superiore Di Sanità, Rome, Italy; 2 European Reference Laboratory for Parasites, Department of Infectious Diseases, Istituto Superiore Di Sanità, Rome, Italy; 3 Department of Biology, Ethology Unit, University of Pisa, Pisa, Italy; 4 Department of Ecosystem and Public Health, Faculty of Veterinary Medicine, University of Calgary, Calgary, AB Canada; 5 UMR CNRS 6249 Chrono-Environnement, Université Bourgogne Franche-Comté, Besancon, France; 6 Department of Zoology, Institute of Ecology and Earth Sciences, University of Tartu, Tartu, Estonia; 7 Anses LRFSN, National Reference Laboratory for Echinococcus Spp, Malzéville, France

**Keywords:** *Echinococcus granulosussensu lato*, *Echinococcus granulosussensu stricto*, *Echinococcus canadensis*, *Echinococcus ortleppi*, Genotypes, Human cystic echinococcosis, Europe

## Abstract

**Background:**

This study aimed to fill a gap of knowledge by providing a quantitative measure of molecularly identified species and genotypes belonging to *Echinococcus granulosus* *sensu lato* (*s.l.*) causing human cystic echinococcosis (CE) in Europe during the period 2000–2021. As these species and genotypes are characterized by genetic, animal host and geographical differences, studying the *E. granulosus* *s.l.* complex is epidemiologically relevant.

**Methods:**

A systematic review (SR) was conducted on the basis of both scientific and grey literature considering primary studies between 2000 and 2021 in four databases. From a total of 1643 scientific papers, 51 records were included in the SR. The main inclusion criterion for this study was the molecular confirmation of *E. granulosus* *s.l.* at the genotype/species level as a causative agent of human CE cases in selected European countries.

**Results:**

Relevant data were obtained from 29 out of 39 eligible European countries. This SR identified 599 human molecularly confirmed echinococcal cysts: 460 (76.8%) identified as *E. granulosus* *sensu stricto* (*s.s.*), 130 (21.7%) as *E. canadensis* cluster (G6/7 and G10), 7 (1.2%) as *E. ortleppi* (G5), and 2 as *E. vogeli* (0.3%). Three geographical hotspots of human CE caused by different species of the *E. granulosus* *s.l.* complex were identified: (1) *E. granulosus* *s.s.* in Southern and South-eastern Europe (European-Mediterranean and Balkan countries); (2) *E. canadensis* (G6/7) in Central and Eastern Europe; (3) *E. ortleppi* in Central and Western Europe. This SR also identified data gaps that prevented a better definition of the geographical distribution of the *Echinococcus granulosus* *s.l.* species complex in Europe: western Balkan countries, part of Central Europe, and Baltic countries.

**Conclusions:**

These results mandate longitudinal, multi-centre, intersectoral and transdisciplinary studies which consider both molecular and clinical epidemiology in animals and humans. Such studies would be valuable for a better understanding of the transmission of the *E. granulosus* *s.l.* species complex and their potential clinical impact on humans.

**Graphical Abstract:**

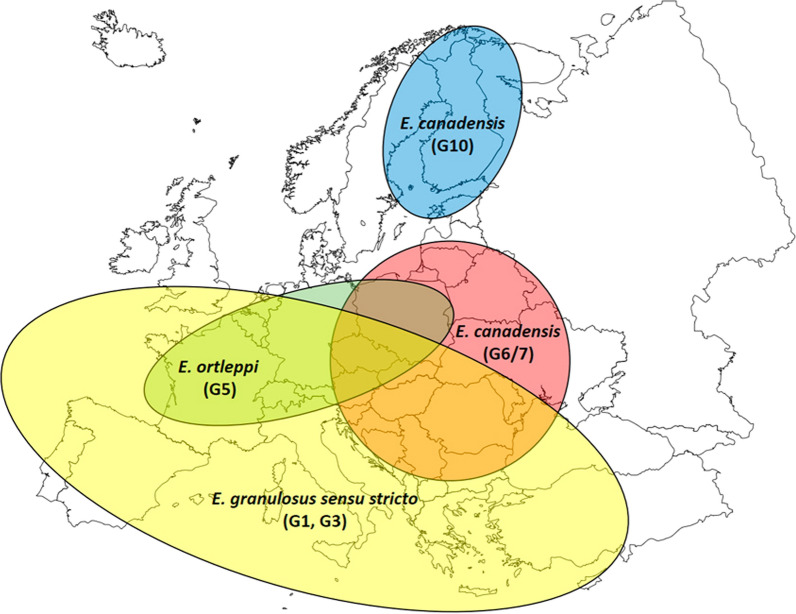

**Supplementary Information:**

The online version contains supplementary material available at 10.1186/s13071-022-05197-8.

## Background

According to the World Health Organization (WHO) criteria for classification, “echinococcosis” human infections fall among the neglected diseases, disorders or conditions of global health importance for which WHO advocates their control [1]. “Echinococcosis” is a parasitic disease group which includes cystic (CE), alveolar (AE) and neotropical (NE) echinococcosis, with more than one million people estimated to be infected at any one time [2, 3]. Among *Echinococcus* spp. infections, CE is the most prevalent at the global level, causing high morbidity and relative mortality among human populations [4].

Causative agents of echinococcosis are endoparasitic tapeworms belonging to *Echinococcus* genus, which is currently divided into the following species: *Echinococcus granulosus* *sensu lato* species complex, *Echinococcus multilocularis*, *Echinococcus shiquicus*, *Echinococcus vogeli* and *Echinococcus oligarthra* [5]. *Echinococcus granulosus* *s.l.* is the complex of cryptic species and genotypes causing CE in humans and animals [6]. The life cycles of *Echinococcus* species are indirect and involve two mammalian hosts: an intermediate (IH) and definitive (DH) host, which are connected in the trophic chain.

During the last decades, molecular analysis based principally on sequencing of mitochondrial DNA clarified the extent of genotypes, showing differences in lineages, geographical distribution and animal host variability. On the basis of phylogenetic analysis and parasite host specificity in their life cycles, five species belonging to the *E. granulosus* *s.l.* complex are currently recognized [7]: *Echinococcus granulosus* *sensu stricto*, *Echinococcus equinus*, *Echinococcus ortleppi*, *Echinococcus canadensis* and *Echinococcus felidis*.

*Echinococcus granulosus* *s.s.* includes genotypes G1 and G3, formerly described as “sheep strain” and “buffalo strain”, respectively. Genotype G2 is no longer considered a valid genotype, but it is recognized as a microvariant of G3 [8]. *Echinococcus granulosus* *s.s.* is distributed worldwide owing to both low IH specificity and extended livestock trade. Main IH and DH contributing to the maintenance of the parasite life cycle are small ruminants (particularly sheep) and dogs (mostly shepherd dogs), respectively. Among *E. granulosus* *s.l.* species, *E. granulosus* *s.s.* is the most relevant species of public health importance since it causes 88.5% of worldwide documented human CE infections [9].

*Echinococcus equinus* is represented by genotype G4, formerly described as “horse strain”. Main IH and DH contributing to the maintenance of the parasite life cycle are equids and dogs. This species was thought not to be zoonotic for long until two cases of human infection were recently documented in Turkey and Uzbekistan [10, 11].

*Echinococcus ortleppi* is represented by genotype G5, formerly described as “bovine strain”. Main IH and DH contributing to the maintenance of the parasite life cycle are cattle and dogs. Thanks to increased hygiene practices in cattle breeding and slaughtering, this parasitic infection has become rare in animals and few human infections have been documented worldwide [9].

*Echinococcus canadensis* cluster is actually divided in two main clades: genotypes G6/7 (formerly described as “camel strain” and “pig strain”, respectively) and genotypes G8/G10 (also referred to as “cervid strains”). Genotypes G6/7 are distributed worldwide and have quite low IH specificity, involving predominantly domestic species such as pigs, camels and goats as IH and dogs as DH. Within the *E. granulosus* *s.l.* complex, *E. canadensis* (G6/7) is the second most relevant species of public health importance, causing around 11% of worldwide documented human CE infections [9]. Genotypes G8/G10 have circumpolar distribution in the Northern Hemisphere. Main IH and DH contributing to the maintenance of the parasite life cycle are wild cervids (such as moose and deer) and wolves, respectively, but to a lesser extent also semi-domestic reindeer and hunting, sledding or shepherd dogs. Few *E. canadensis* (G8/G10) human infections have been documented so far. While in this manuscript the authors will adopt the above-mentioned classification, it should be noted that the taxonomic status of *E. canadensis* cluster (G6/7, G8 and G10) is still under debate [12]. In fact, several new species have been proposed, but not yet accepted internationally (*Echinococcus intermedius* for G6/7, *Echinococcus borealis* for G8 and *Echinococcus canadensis* for G10) [2].

The last species of the *E. granulosus* *s.l.* complex, *E. felidis* (also referred to as “lion strain”), has a wildlife cycle and is only present in sub-Saharan Africa. No human infections have been documented so far.

In this epidemiological context, this systematic review (SR) aimed to fill a gap of knowledge by providing an exhaustive overview on species and genotypes of *E. granulosus* *s.l.* infecting humans and that circulate or were imported in Europe. The primary aim of this SR was to quantify the total number of molecularly identified human cases and to map the distribution of genotypes and species causing human CE infections by country in Europe from 2000 to 2021. The secondary aim of this SR was to identify gaps in knowledge of genotype/species circulating in specific European geographical areas.

## Methods

This SR was conducted according to PRISMA Group guidelines [13] (Additional file 1: Table S1). The systematic search was carried out using the Documentation Service for literature search at the Istituto Superiore di Sanità, Rome, Italy. The STN International-Fiz Karlsruhe platform [14] was used for database searching carried out on 14 May 2021 to identify articles that had been published since the initial search. The databases screened in the literature search were MEDLINE (PubMed), Embase (Excerpta Medica Database), SciSearch (Science Citation Index) and Google Scholar. Databases were searched using keywords associated with the Boolean operators AND and OR. The full electronic search strategy, including any limits used, was: (“cystic echinococcosis” OR Hydatid* OR echinococcal OR Echinococcus OR E* granulosus OR E* canadensis OR E* equinus OR E* ortleppi) AND (OR Human OR children OR teenager OR child OR boy OR girl OR young) AND (Europe OR “European Union” OR European) NOT (alveolar OR multilocularis OR E. multilocularis OR “Echinococcus multilocularis”) NOT (“hydatid mole” OR “hydatidiform mole”).

In the first search carried out on the STN International-Fiz Karlsruhe platform, only papers in English published between 2000 and 2021 were included in the SR. A second search was conducted till 4 February 2022 without any language restriction for the identification of papers, reports, datasets or other grey literature from countries where no data or little data were identified in the first search. The bibliography of articles found in both these searches was assessed for additional records. Experts in this field were also contacted by email from the network of the National Reference Laboratory (NRL) for Parasites (https://www.iss.it/en/web/iss-en/eurlp-about-us) and from additional scientific networks for the identification of data not published in the scientific literature. Such data were inserted in this SR as *personal communication* (PC).

The main inclusion criterion for this study was the molecular confirmation of *E. granulosus* *s.l.* at the genotype/species level as a causative agent of human CE infections. Studies eligible for inclusion were human case reports, case series, epidemiological investigations or datasets reporting the molecular identification by mitochondrial or nuclear genes of genotypes or species belonging to the *E. granulosus* *s.l. *complex in Europe within the period 2000–2021. Studies were excluded if they lacked original data (e.g. reviews not containing primary data) or duplicated data (e.g. between papers and other records) or involved the wrong aetiologic agent (e.g. *Echinococcus multilocularis*) or infectious/not infectious disease (e.g. alveolar echinococcosis or hydatid mole) or host (e.g. intermediate or definitive animal host). No restrictions in the search was posed on animals since some epidemiological studies on molecular identification of *E. granulosus* *s.l.* also contained human data. Countries included in this search were all those within the European borders, including Albania, Austria, Belarus, Belgium, Bosnia and Herzegovina, Bulgaria, Croatia, Czech Republic, Denmark, Estonia, Finland, France, Germany, Greece, Hungary, Italy, Ireland, Latvia, Lithuania, Luxembourg, Malta, Montenegro, Norway, Poland, Portugal, Republic of Cyprus, Republic of North Macedonia, “Republic of Kosovo”, Republic of Moldova, Romania, Serbia, Slovak Republic, Slovenia, Sweden, Switzerland, Spain, the Netherlands, the UK and Ukraine.

Duplicates between databases were removed, and the study selection process was carried out by two independent researchers for the selection of papers to be included in this SR. Any disagreement was resolved by consensus between the two researchers. An initial screening was undertaken according to the title and abstract’s relevance in terms of the focus of this study. The full texts of the shortlisted papers were examined through a second screening stage to assess their eligibility. Data were extracted from eligible papers and entered into standardized Microsoft Excel tables (Microsoft Office, 2016). For each retained paper, the following data were extracted into tables: the reference article, country where human CE cases were detected, country of origin (nationality) of human CE case, human CE cases assumed to be locally acquired or imported (according to case-definition of this study given below), species and genotypes (as reported by the authors of the studies) of *E. granulosus* *s.l.* When a human CE case was attributed to *E. granulosus* *s.s. *without further discrimination between G1 and G3 genotypes, it was recorded as “ungenotyped”.

The nationality of patients was used as a proxy to define whether CE cases were presumably imported or not, since these patients were mostly migrants from highly endemic countries for CE. When the nationality of a patient was different from that expected in the country where the CE diagnosis was made, the infection was considered as not locally acquired. In particular, when a specific *Echinococcus* species was imported in a given European country from another European country eligible in this study, it was considered as circulating between countries in Europe and such human cases were recorded in the country of origin. When a specific *Echinococcus* species was detected in a European country from patients of non-European nationality, such human cases were considered as imported in Europe.

## Results

**Fig. 1:**
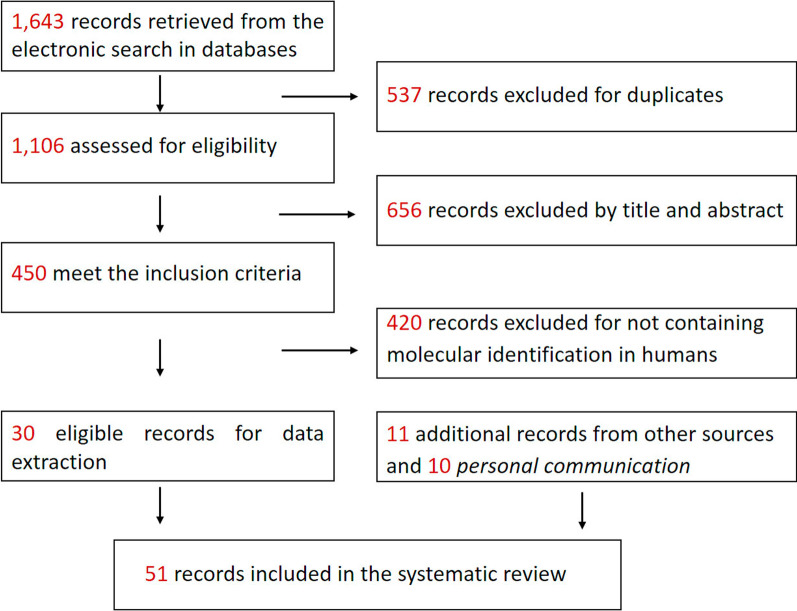
Flow chart representing the algorithm applied to select articles from the databases

The literature search identified a total of 1643 potentially relevant papers, from which 537 duplicates were excluded (Fig. 1), resulting in 1106 papers assessed for eligibility. Subsequently, 656 papers were excluded by checking the title and abstract. Therefore, text of 450 papers was assessed for inclusion criteria. Subsequently, 420 full-text papers were excluded for not containing molecular identification of *E. granulosus* *s.l.* in humans during the considered time period. Data were extracted from a total of 41 eligible papers resulting from the two searches [15–55]. Additional records were identified from other sources, including *personal communications* (*pers comm*) from European experts in this field [*n* = 10; Peter Deplazes *pers comm* (PC1) on 26/11/2021, Relja Beck *pers comm* (PC2) on 28/11/2021, Famke Jansen *pers comm* (PC3) on 2/12/2021, Jenny Knapp *pers comm* (PC4) on 3/12/2021, Øvind Øines *pers comm* (PC5) on 2/12/2021, Cinzia Santucciu *pers comm* (PC6) on 9/12/2021, Simona Gabrielli *pers comm* (PC7) on 13/12/2021, Marion Wassermann *pers comm* (PC8) on 14/12/2021, Tamás Sréter *pers comm* (PC9) on 01/02/2022 and Smaragda Sotiraki *pers comm* (PC10) on 04/02/2022]. Finally, a total of 51 records were included in the SR for data extraction (Fig. 1).

Relevant data on genotype and species identification in humans were obtained from 29 eligible countries [20 were European Union (EU) member states], namely: Albania, Austria, Belgium, Bosnia and Herzegovina, Bulgaria, Croatia, Finland, France, Germany, Greece, Hungary, Italy, Lithuania, Luxembourg, Norway, Poland, Portugal, Republic of North Macedonia, “Republic of Kosovo”, Republic of Moldova, Romania, Serbia, Slovak Republic, Slovenia, Switzerland, Spain, the Netherlands, the UK and Ukraine. Data where searched but not obtained from the following ten eligible countries (eight were EU member states): Belarus, Czech Republic, Denmark, Estonia, Ireland, Latvia, Malta, Montenegro, Republic of Cyprus, and Sweden.

Additional file 2: Table S2 summarizes data extraction on the countries of origin of CE patients, countries were these human CE cases were diagnosed, the identification of the causative *Echinococcus* species and/or genotypes, and if these specimens were assumed to be locally acquired or not.

This SR identified a total of 599 human CE cases that were molecularly identified in European studies (Additional file 2: Table S2; Table 1). A total of 413 (68.9%) human echinococcal cysts were identified as *E. granulosus* *s.l.* circulating between countries in Europe, 12 (2%) belonged to patients of unknown country of origin (no data available for these CE cases in France, Germany, Norway or Slovenia) and 78 (13%) belonged to patients of unknown imported country of origin (it was only known that these CE cases were imported in Belgium, Finland, Germany, Luxembourg or Switzerland), while 96 (16%) were imported from non-European countries.

**Table 1:** Number of CE cases belonging to *E. granulosus sensu lato* complex species at country level in Europe

	Total human CE cases detected in the study (*n* = 599)									Human CE cases circulating in Europe (*n* = 413)						
	*E. granulosus s.l.* (total)	*E. granulosus s.s.* (G1, G3)	%	*E. ortleppi* (G5)	%	*E. canadensis* (G6/7, G10)	%	*E. vogeli*	*E. granulosus s.l.* (total)	*E. granulosus s.s.* (G1, G3)	%	*E. ortleppi* (G5)	%	*E. canadensis* (G6/7, G10)	%	References***
Albania	3	3	100						3	3	100					32, 45, PC7
Austria	77	50	64.9			27	35.1		33	9	27.3			24	72.7	32, 46
Belgium	1	1	100													PC3
Bosnia and Herzegovina	5	5	100						5	5	100					45, PC8
Bulgaria	41	41	100						41	41	100					19, 29, 32, 42
Croatia	6	6	100						6	6	100					43, PC2
Finland	3	2	66.7			1*	33.3		1					1*	100	26, 28, 42
France	37	30	81.1	4	10.8	3	8.1		10	6	60	4	40			25, 40, 41, PC4
Germany	68	66	97.1	1	1.5	1	1.5									51, PC8
Greece	2	2	100						2	2	100					32, PC10
Hungary	3	2	66.7			1	33.3		3	2	66.7			1	33.3	32, 44, PC9
Italy	48	48	100						42	42	100					17, 38, 32, 39, 51, 53, PC6, PC7, PC8
Lithuania	3					3	100		3					3	100	50
Luxembourg	1					1	100									PC4
Norway	1	1	100													PC5
Poland	68	2	2.9	1	1.5	65	95.6		67	1	1.5	1	1.5	65	97	22, 23, 24, 31, 44, 47, 54
Portugal	1	1	100						1	1	100					16
Republic of North Macedonia	3	3	100						3	3	100					45, PC8
“Republic of Kosovo”	1	1	100						1	1	100					PC4
Republic of Moldova	16	15	93.8			1	6.3		16	15	93.8			1	6.3	36, PC4
Romania	76	74	97.4			2^+^	2.6		76	74	97			2^+^	2.6	15, 30, 37, 32, 42, 44, 51, 53, PC4, PC7
Serbia	9	6	66.7			3	33.3		9	6	67			3	33	21, 49, PC8
Slovak Republic	6	2	33			4	67		6	2	33			4	67	35, 44, 55
Slovenia	6	1	16.7			5	83.3		5					5	100	45
Switzerland	33	29	87.9	1	3	3	9.1		1			1	100			PC1
Spain	46	46	100						46	46	100					20, 28, 42, 48, 53, PC8
The Netherlands	2							2								33, 34
UK	4	4	100						4	4	100					18
Ukraine	2	1	50			1	50		2	1	50			1	50	44, PC7
Ex Yugoslavia (Bosnia, Serbia, Kosovo, Macedonia) **	27	18	66.7			9	33.3		27	18	66.7			9	33.3	32
Total	599	460		7		130		2	413	288		6		119		
Percentages		76.8%		1.2%		21.7%		0.3%		69.7%		1.5%		28.8%		

On the left, total number of cases detected in Europe, including those imported from non-European countries. On the right, only cases circulating in Europe excluding those imported from non-European countries and those of unknown country of origin

^*^The only case identified as genotype G10 of *E. canadensis*. All other genotypes of *E. canadensis* belong to genotype G6/7

^+^One case from Romania has been generically identified by authors as *E. canadensis* cluster G6/10 [30]

^**^Not possible to separate countries from the reporting study in Austria

^***^The term PC reported among the references stands for *personal communication* from experts in this field. See results for specific information regarding the authors of PC from 1 to 10

Among 413 human CE cases molecularly confirmed in Europe, the nationality of 350 (84.7%) cases coincided with country of diagnosis, whereas for 63 (15.3%) it did not. Considering these 413 human molecularly confirmed CE cases circulating in Europe, 288 (69.7%) were identified as *E. granulosus* *s.s.* [116 (40.3%) genotype G1, 31 (10.8%) genotype G3 and 141 (48.9%) ungenotyped], 119 (28.8%) as *E. canadensis* cluster [117 (98.3%) as G7, 1 (0.85%) as G10, 1 (0.85%) as G6/G10] and 6 (1.5%) as *E. ortleppi* (G5).

CE human cases were of nationality of the following 22 non-European countries: Turkey (*n* = 46), Morocco (*n* = 13), Algeria (*n* = 6), Iraq (4), Tunisia (*n* = 3), China (*n* = 2), Ghana (*n* = 2), Iran (*n* = 2), Kazakhstan (*n* = 2), Lebanon (*n* = 2), Russia (*n* = 2), Suriname (*n* = 2), Afghanistan (*n* = 1), Armenia (*n* = 1), Chile (*n* = 1), Eritrea (*n* = 1), India (*n* = 1), Mali (*n* = 1), Mauritania (*n* = 1), Nigeria (*n* = 1), Syria (*n* = 1) and Thailand (*n* = 1). Among these 96 CE cases from non-European countries, 88 (91.7%) were identified as *E. granulosus* *s.s.*, while 6 (6.2%) as *E. canadensis* [3 genotypes G6 from Iran (*n* = 1), Afghanistan (*n* = 1) and Ghana (*n* = 1) and 3 genotypes G6/7 from Iran (*n* = 1), Mali (*n* = 1) and Mauritania (*n* = 1)] (Additional file 2). This study also identified two cases (2.1%) of patients from Suriname and diagnosed in the Netherlands that were considered CE, but then molecularly identified as *E. vogeli*. These two cases are the first and only NE human cases ever documented in Europe [33, 34] (Additional file 2: Table S2 and Table 1; not included in the figures).

Considering 599 human molecularly confirmed CE human cases identified in this study as a whole, 460 (76.8%) were identified as *E. granulosus* *s.s.* [165 (35.9%) genotype G1, 44 (9.6%) genotype G3 and 251 (54.5%) ungenotyped], 130 (21.8%) as *E. canadensis* cluster [117 (90%) as G7, 1 (0.8%) as G10, 3 (2.3%) as G6, 8 (6.2%) as G6/7 and 1 (0.8%) as G6/G10], 7 (1.2%) as *E. ortleppi* (G5) and 2 as *E. vogeli* (causing NE) (0.3%) (Table 1).

**Fig. 2:**
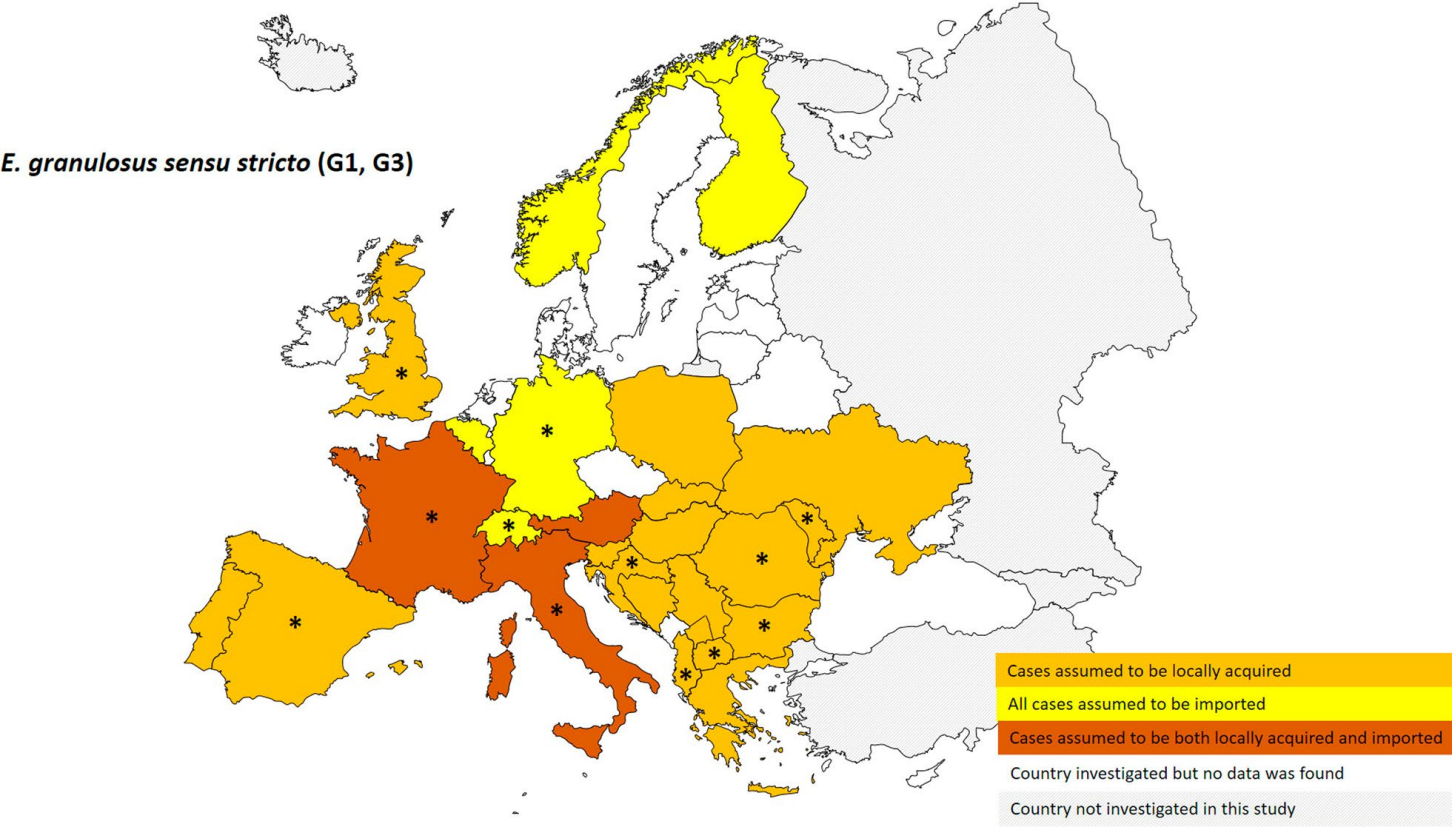
Distribution at European country level of human cystic echinococcosis caused by *Echinococcus granulosus sensu stricto* (G1, G3) (*n* = 460; 2000–2021). * Dominant species in the country (considered when at least three samples were analysed and frequency was > 80%)

**Fig. 3:**
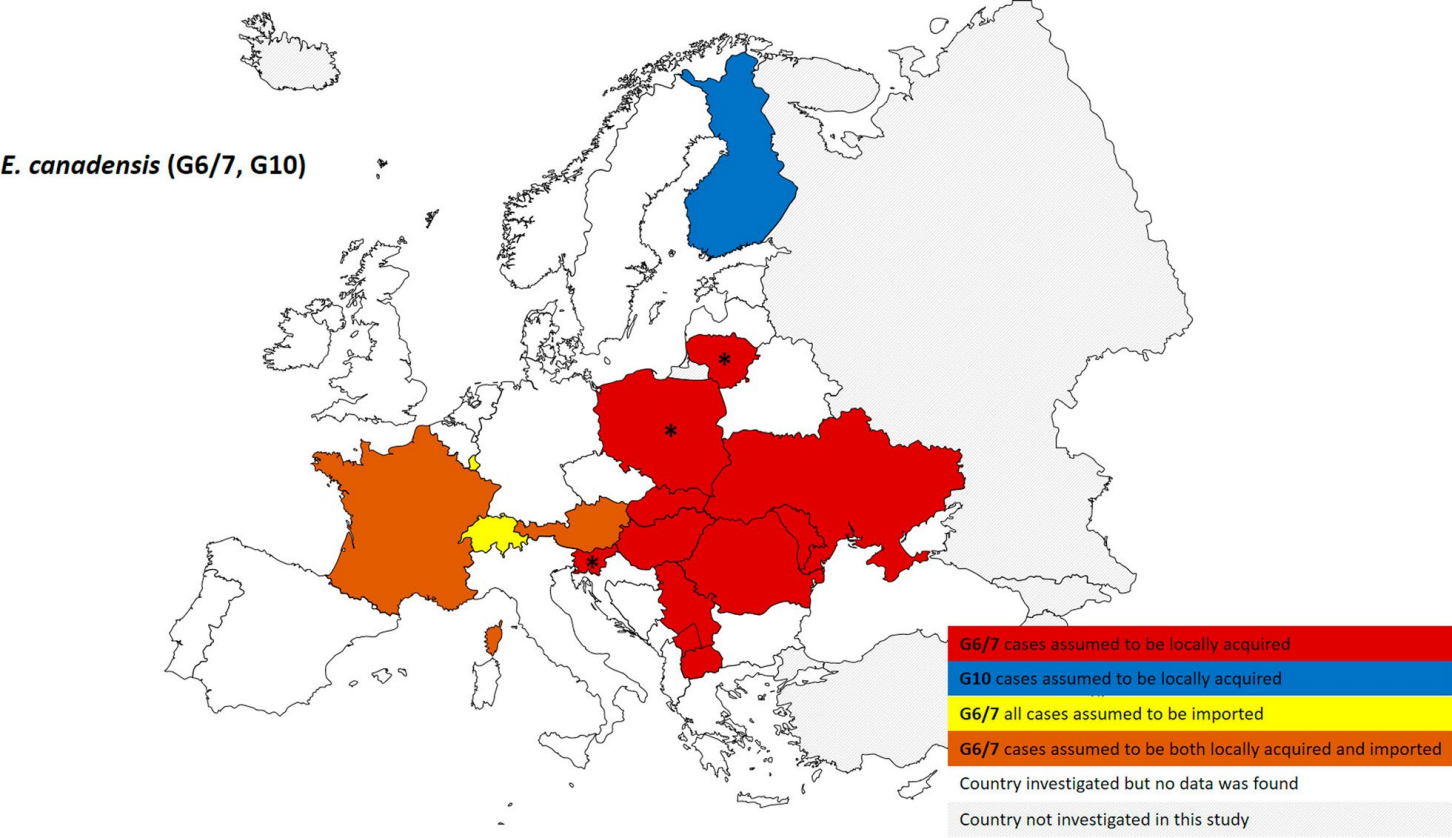
Distribution at European country level of human cystic echinococcosis caused by *Echinococcus canadensis* (G6/7, G10) (*n* = 130; 2000–2021). * Dominant species in the country (considered when at least three samples were analysed and frequency was > 80%)

When considering the presence at European country level of species identified as *E. granulosus* *s.s.* (G1, G3) based on 460 specimens (Fig. 2), Belgium, Finland, Germany, Norway, Poland and Switzerland reported only imported human cases of human CE. Of countries with cases considered only locally acquired or both locally acquired and imported, *E. granulosus* *s.s.* was the dominant species (> 80% when *n* ≥ 3; Table 1) detected in Albania, Bulgaria, Croatia, France, Italy, Republic of Moldova, Republic of North Macedonia, Romania, Spain and the UK. We identified Southern and South-eastern Europe (European-Mediterranean and Balkan countries) as hotspots of human CE caused by *E. granulosus* *s.s.*; specifically, in areas where sheep breeding is widely practised at large-/small-scale farms (Fig. 5). Figure 3 shows the presence at European country level of species identified as part of the *E. canadensis* cluster (genotypes G6/7 and G10; no human infections due to genotype G8 have been reported in Europe) based on 130 specimens. Genotype G10 was only documented in one locally acquired case from Finland, confirming rareness of this species infection in humans. Luxembourg and Switzerland, considered non-endemic countries for CE, were the only countries reporting only imported cases of genotype G6/7. Considering countries with cases considered only locally acquired or both locally acquired and imported, *E. canadensis* G6/7 was the dominant *Echinococcus* species (> 80% when *n* ≥ 3; Table 1) detected in Lithuania, Poland and Slovenia. We identified a hotspot of human CE caused by genotype G6/7 of *E. canadensis* in Central and Eastern Europe, typically areas where non-intensive pig raising is commonly practised (Fig. 5).

**Fig. 4:**
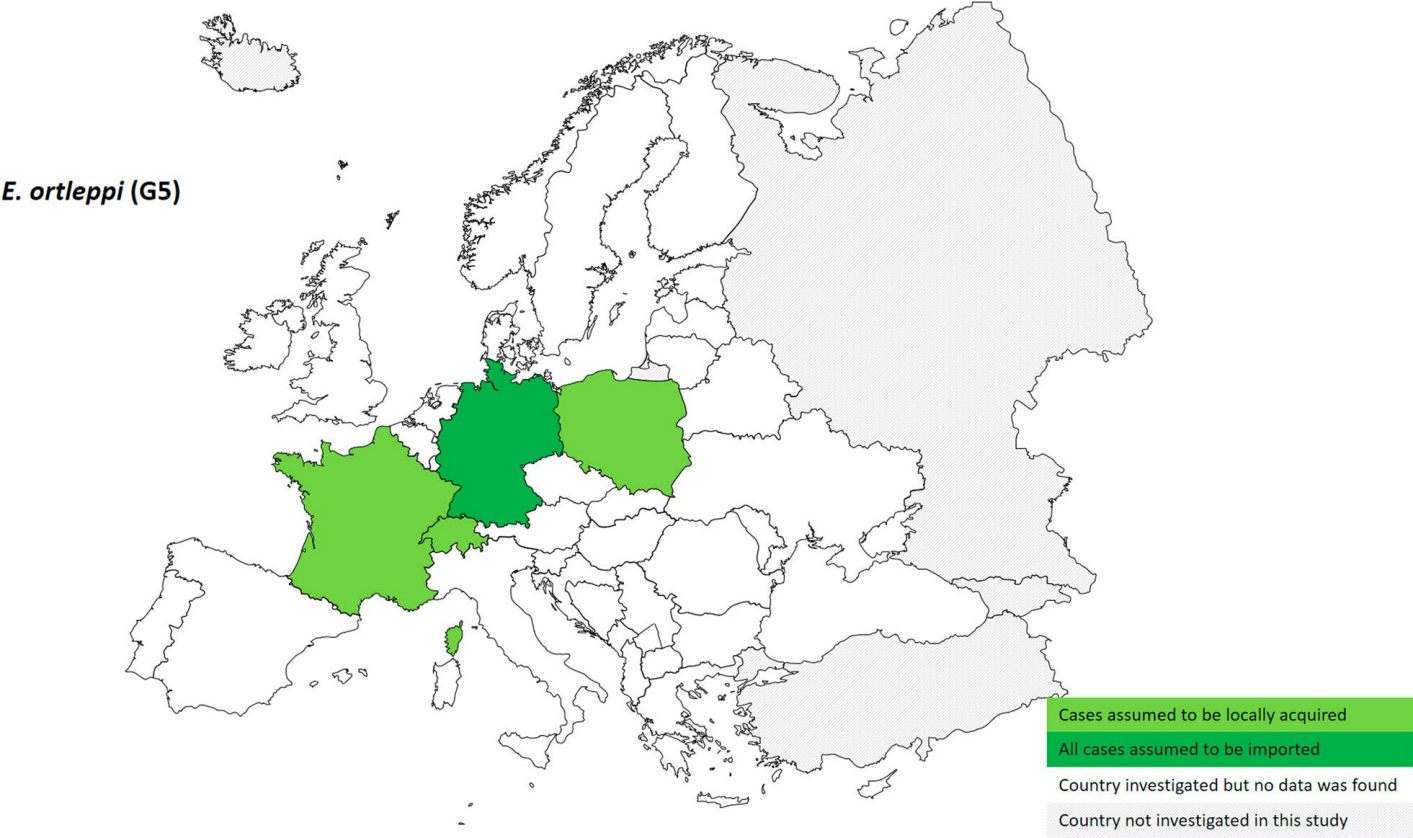
Distribution at European country level of human cystic echinococcosis caused by *Echinococcus ortleppi* (G5) (*n* = 7; 2000–2021)

**Fig. 5:**
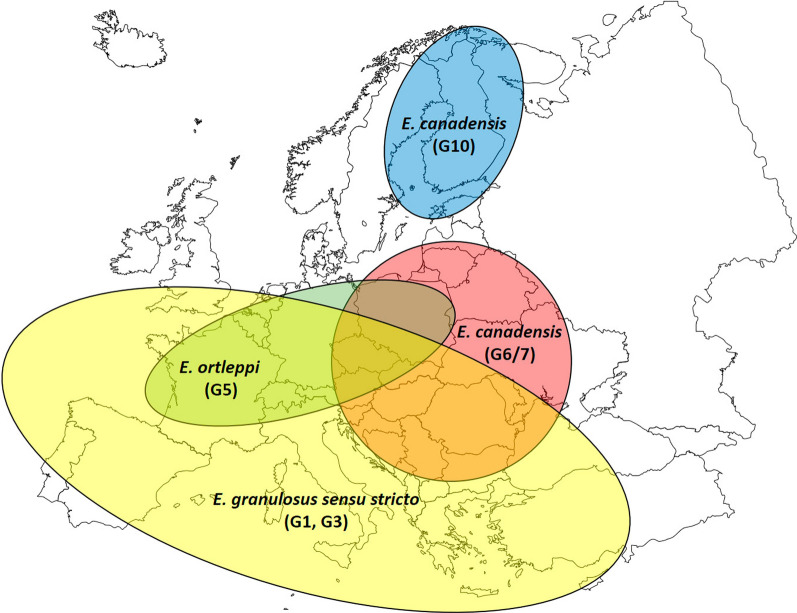
Approximate geographical distribution of *Echinococcus granulosus sensu lato* species complex causing human cystic echinococcosis in Europe (2000–2021) according to 599 molecularly identified parasitic cysts. Two cysts were identified as *E. vogeli* causing neotropical echinococcosis and were therefore excluded from this map

The presence at European country level of species identified as *E. ortleppi* (G5) was determined on the basis of seven specimens, therefore confirming the rareness of this species infection in humans (Fig. 4). During the past 20 years, infections caused by *E. ortleppi* were assumed to be locally acquired only in France (*n* = 4), Poland (*n* = 1) and Switzerland (*n* = 1). Germany documented one imported case of unknown country of origin. This figure identifies Central and Western Europe as hotspots for the few human CE infections caused by *E. ortleppi*, areas where cattle raising is practised (Fig. 5).

## Discussion

This SR provides the first comprehensive view and synthesis of the genotypes and species belonging to the *E. granulosus* *s.l.* complex infecting humans in Europe during the period 2000–2021. A previous valuable attempt was done by Alvarez Rojas and colleagues in 2014, who identified 1661 molecularly confirmed human CE cases at global level, 188 (11.3%) of which were from 16 European countries [9]. In this review, 1474 cases (88.44%) were identified as *E. granulosus* *s.s.* (all 131 European samples overlapping with our study), 184 (11.07%) as *E. canadensis* G6/7 (125 European samples overlapping with our study, one not since it was detected before 2000), 6 (0.36%) as *E. ortleppi* (all cases not overlapping with our study: 5 from non-European countries and 1 detected in the Netherlands before 2000), 1 (0.06%) as *E. canadensis* genotype G8 (genotype not identified in our study) and 1 (0.06%) as *E. canadensis* G10 (case from Asia, not overlapping with our case from Finland).

In line with the review by Alvarez-Rojas and colleagues [9], the current SR in Europe has confirmed *E. granulosus* *s.s.* as the main driver of CE infections in humans. However, the proportion of cases attributed to *E. canadensis* (G6/7) in Europe is almost double that detected worldwide (21.8% versus 11.1%) [9]. It should be noted that the frequency of *E. canadensis* G6/7 in this study, as well as that at the worldwide scale, could be biased by sampling effort. In fact, the number of molecularly confirmed human samples probably does not reflect the actual numerical burden of CE in specific endemic countries (e.g. countries that account for the majority of human cases in Europe, such as Italy and Spain, where *E. granulosus* *s.s.* is the dominant species), but rather the presence of experts in those countries [56, 57]. However, a higher burden than expected of *E. canadensis* G6/7, but also other species and genotypes, in Europe cannot be ruled out.

This study identified seven human cases infected with *E. ortleppi*: six considered as locally acquired in France, Poland and Switzerland, and one considered as imported in Germany (23, 25, 40, 51, PC1, PC4). To the best of our knowledge, 16 additional CE cases (23 in total) caused by *E. ortleppi* have been detected in humans globally from Argentina (*n* = 5), Brazil (*n* = 1), China (*n* = 2), India (*n* = 1), Mexico (*n* = 1), the Netherlands (*n* = 1), South Africa (*n* = 1) and Vietnam (*n* = 4) [58–69].

Our study identified only one locally acquired case of *E. canadensis* genotype G10 from Finland [26]. To the best of our knowledge, only four additional CE cases (11 in total) caused by G10 have been detected in humans globally from China (*n* = 1), Far East Russia (*n* = 1) and Mongolia (*n* = 2) [70–73]. No case of *E. canadensis* genotype G8 was identified in this study from Europe. Only one human infection has been globally documented in the past from Alaska [74].

The main drivers of infection pressure of species of *E. granulosus* *s.l.* on humans in Europe may reflect: (1) the presence of high numbers of the main IH (e.g. sheep for *E. granulosus s.s.*, pigs for *E. canadensis* G7, cattle for *E. ortleppi*, wild ungulates for *E. canadensis* G8 and G10), which maintain the parasite species life cycles and consequently the biomass of viable eggs contaminating the environment; (2) the hygiene conditions of rearing animals (e.g. home slaughtering versus controlled slaughtering) and type of husbandry (e.g. intensive versus backyard practices), which may increase odds of transmission of *Echinococcus* species to humans; (3) deworming practices of dogs DH that release eggs, the parasite infective stage for humans. In this context, the few human infections detected in Europe caused by *E. ortleppi* and *E. canadensis* G10 may be explained by the above-mentioned low infection pressure of these *Echinococcus* species. In fact, the improved hygiene conditions of cattle breeding during past decades in Europe (with dogs not having access to their offal) decreased the presence of *E. ortleppi* in animal hosts (e.g. the disappearance of this parasite species in Switzerland). On the same note, the sylvatic cycles of genotypes G8 and G10 may explain the low exposure of humans and, consequently, the few documented human infections.

The potentially different infectivity and pathogenicity of the *E. granulosus* *s.l.* species to humans has already been debated by several authors [32, 59, 75, 76]. Whether these genotypes and species may be of different grade of infectivity and pathogenicity to humans (namely different speed and size of growth, and anatomical site predilection of echinococcal cysts) is currently not clear, and requires further investigation by means of systematic studies implementing large cohorts of patient, including comparators (control groups). According to current knowledge, the molecular identification of the species/genotypes of *E. granulosus* *s.l.* is not relevant in daily clinical practice, as it does not influence the clinical management, treatment and follow-up of human CE cases. Nevertheless, it must be pointed out that the identification of genotypes and species of *E. granulosus* *s.l.* circulating in humans is important in identifying the reservoir species of the parasite circulating in humans, and consequently the possibility of assessing source attribution and risk factors that should be targeted for implementing surveillance and control programmes. Moreover, different parasite biological potential due to different prepatent periods of worms in the DH and potential difference in efficacy of EG95 vaccine in the IH should be considered [9, 77].

Finally, it is worth noting two potential limits of this study, namely the assumption about the nationality of the patients and the identification of *E. granulosus* *s.s.* and *E. canadensis* genotypes, as reported by the authors of the studies included in this SR. The limit of nationality, the time lag (months/years) between the event of *Echinococcus* spp. infection and the eventual appearance of symptoms make it almost impossible to trace the source and place of human CE infection. Assuming this level of uncertainty, the nationality of patients was used as a proxy to define the country of infection, i.e., if these *Echinococcus* species/genotypes were circulating or not in a given European country. As for the study of Alvarez Rojas [9] (see country of origin of Austrian patients), the nationality of patients was used in this SR to define whether CE cases were presumably imported or not, since these patients were mostly migrants from highly endemic countries for CE where specific genotype/species are expected. As an example, this SR identified 68 molecularly confirmed human samples diagnosed in Poland, 65 of which were found to be *E. canadensis* (G7), 1 *E. ortleppi* and only 2 *E. granulosus* *s.s.* In Poland, *E. granulosus* *s.s.* was identified for the first time in 2017 in only one sheep [31], while *E. canadensis* G7 has been widely documented in animals and humans [22–24, 31, 47]. In this context, among the only two human patients diagnosed in Poland as *E. granulosus* *s.s.*, the first was of Kazak nationality (highly endemic areas for *E. granulosus* *s.s.*, where this patient was previously operated), and the second had a history of travel 2 years before in Turkey (another highly endemic country for *E. granulosus* s.s). According to our case definition, the first case was considered not originated in Europe but in Kazakhstan, while the second, though the patient was probably exposed in Turkey, was considered as locally acquired in Poland [22]. None of the other samples associated with nationality resulted in an unexpected genotype or species in a specific county (Table 1; Additional file 2). With regard to the limit of the identification of genotypes (G1, G3, G6, G7, G6/7 and ungenotyped), we recorded this information as reported by authors of the studies included in the SR. This classification cannot always be verified because of either the absence of sequences deposited in databases, the molecular method implemented or the short sequence analysed by authors that may compromise the correct identification of genotypes in light of more recent studies on genotyping [8, 12].

## Future perspective

Having illustrated the relevance of collecting molecular data, the standing question is what path clinicians should follow when handling parasitic samples from CE cases. The authors suggest for this task to refer to the NRL for Parasites for *E. granulosus* *s.l.* species molecular identification. In the European Union, a network of 41 NRL for Parasites is present, and in support of them, the European Union Reference Laboratory for Parasites (EURLP; https://www.iss.it/web/iss-en/eurlp-about-us) can be contacted to coordinate these analyses (Commission Regulation 776/2006).

Moving towards a standardized genotyping approach to be integrated into the national public health system is the way forward. During the past 30 years, molecular typing data of *E. granulosus* *s.l.* species have largely been generated on the basis of mitochondrial *cox1* gene sequencing [58]. Recently, a much more sensitive real-time PCR assay [78] and a validated method not requiring sequencing [79] to differentiate *E. granulosus* *s.l.* species have been made available in literature, as well as new molecular tools for genotype differentiation (G1 versus G3 and G6 versus G7) within this species complex [80, 81].

Finally, we encourage longitudinal, collaborative, multi-centre, intersectoral and transdisciplinary research in Europe and beyond to perform molecular analysis on *Echinococcus* isolates, keeping together molecular and clinical epidemiology in animals and humans. Strengthening such studies will lead to a better understanding of the transmission of the *E. granulosus* *s.l.* species complex and their potential clinical impact to humans.

## Conclusions

This SR provided new data at the diversity and distribution of *E. granulosus* *s.l. *species and genotypes infecting humans in Europe. The study also identified a series of gap of knowledge at the country level on the presence and distribution of *E. granulosus* *s.l.* in humans. In particular, no genetic data were available for several endemic or importing countries (Belarus, Czech Republic, Denmark, Estonia, Ireland, Latvia, Malta, Montenegro, Republic of Cyprus, Sweden) or the data were scanty (Albania, Belgium, Bosnia and Herzegovina, Croatia, Finland, Greece, Hungary, Lithuania, Luxembourg, Norway, Portugal, Republic of North Macedonia, “Republic of Kosovo”, Serbia, Slovak republic, Slovenia, the Netherlands, the UK and Ukraine). Moreover, this SR identified two grey zones to better define the geographical distribution of the *E. granulosus* *s.l.* complex in Europe: one overlapping between the distribution of *E. granulosus* *s.s.* and *E. canadensis* G6/7 and the other in the northern European geographical distribution of *E. canadensis* G6/7, where it is not clear which are the main species of *E. granulosus* *s.l.* infecting humans. The first grey zone corresponds to the western Balkan countries (in particular Bosnia and Herzegovina, Republic of North Macedonia, Montenegro, Kosovo and Serbia) and part of Central Europe (in particular Czech Republic and Slovakia), whereas the second zone was identified in the Baltic countries (in particular Latvia and Estonia), where more sampling effort is needed to understand which parasitic species represents a public health problem, resulting in targeted control measures. An additional advance in this direction would be represented by the systematic implementation of shared molecular tools (using dedicated loci) in the field of epidemiology for the molecular identification of causative species belonging to *E. granulosus* *s.l.*

## Supplementary Information

**Additional file 1: Table S1.** PRISMA 2020 Checklist.

**Additional file 2: Table S2.** Summary of data extracted from publications reviewed in this study.

## Data Availability

Materials described in the manuscript, including all relevant raw data, are freely available.
